# Supplementary material for: Cross-modal representation of chewing food in posterior parietal and visual cortex
Source: PLoS One. 2024 Oct 25;19(10):e0310513. doi: 10.1371/journal.pone.0310513 (PMC11508057; doi:10.1371/journal.pone.0310513)
Supplement: Attachment — Submitted filename: Response to Reviewers.docx [file pone.0310513.s002.docx]

**Academic Editor**

**As you will see below, and in accordance with my own assessment of the manuscript, both reviewers raise concerns about the small sample size and the correctness of the reported power analysis. I agree with the reviewers that not enough details are provided to replicate the power analysis in G*Power, but it appears unlikely that a sample size of n=3 would be sufficient to detect a medium-sized effect with 80% power. Please add sufficient details and double-check the correctness of the power calculation. In addition, I agree with Reviewer #2 that the small sample size of n=9 should be discussed as a limitation of the study. Please also address the additional points raised in the reviews, in particular make sure to add the methodological details recommended by Reviewer #2.**

**Response**

**We appreciate the reviewer for pointing this out. In the original version, the number of measurements was incorrect. We were mistaken about the number of measurements. That has been corrected to 25 measurements (5 times repeated, 5 tasks) and the sample size was then recalculated using the following parameters: effect size = 0.3, α = 0.05, power (1-β) = 0.95, number of groups = 1, number of measurements = 25 (5 times repeated, 5 tasks). The sample size required was 9 and the power of this calculation was determined to be 0.974. The modified the Subjects section and the explanation regarding sample size has been added to the Discussion section in revised version.**

**Journal Requirements:**

**1. When submitting your revision, we need you to address these additional requirements.**

**Please ensure that your manuscript meets PLOS ONE's style requirements, including those for file naming. The PLOS ONE style templates can be found at 
https://journals.plos.org/plosone/s/file?id=wjVg/PLOSOne_formatting_sample_main_body.pdf and 
https://journals.plos.org/plosone/s/file?id=ba62/PLOSOne_formatting_sample_title_authors_affiliations.pdf**

**Response**

**We have verified that the manuscript meets PLOS ONE style requirements (including file naming).**

**2. Thank you for stating in your Funding Statement: 
This work was supported by the Dental Support Co., Ltd. and a JSPS KAKENHI (Grant No. 25463029)**

**Please provide an amended statement that declares all the funding or sources of support (whether external or internal to your organization) received during this study, as detailed online in our guide for authors at http://journals.plos.org/plosone/s/submit-now. Please also include the statement “There was no additional external funding received for this study.” in your updated Funding Statement. Please include your amended Funding Statement within your cover letter. We will change the online submission form on your behalf.**

**Response**

**The statement in the Financial Disclosure has been revised as follows.**

**Financial Disclosure**

**This work was supported by the Dental Support Co., Ltd. and a JSPS KAKENHI (Grant**

**No. 25463029). TU and IK receives a salary by the Dental Support Co., Ltd.. There was no additional external funding received for this study.**

**3. Thank you for stating the following in the Competing Interests section:**

**Takeshi Uchida and Ikuo Kantake are employed by Dental Support Co. Ltd. Neither has any potential conflicts of interest to declare with respect to research, authorship, and/or publication of this article. The remaining authors declare that the research was conducted in the absence of any commercial or financial relationships that could be construed as a potential conflict of interest.**

**We note that one or more of the authors are employed by a commercial company: Dental Support Co. Ltd**

**a. Please provide an amended Funding Statement declaring this commercial affiliation, as well as a statement regarding the Role of Funders in your study. If the funding organization did not play a role in the study design, data collection and analysis, decision to publish, or preparation of the manuscript and only provided financial support in the form of authors' salaries and/or research materials, please review your statements relating to the author contributions, and ensure you have specifically and accurately indicated the role(s) that these authors had in your study. You can update author roles in the Author Contributions section of the online submission form.**

**Please also include the following statement within your amended Funding Statement.**

**“The funder provided support in the form of salaries for authors, but did not have any additional role in the study design, data collection and analysis, decision to publish, or preparation of the manuscript. The specific roles of these authors are articulated in the ‘author contributions’ section.”**

**If your commercial affiliation did play a role in your study, please state and explain this role within your updated Funding Statement.**

**b. Please also provide an updated Competing Interests Statement declaring this commercial affiliation along with any other relevant declarations relating to employment, consultancy, patents, products in development, or marketed products, etc.**

**Within your Competing Interests Statement, please confirm that this commercial affiliation does not alter your adherence to all PLOS ONE policies on sharing data and materials by including the following statement: "This does not alter our adherence to PLOS ONE policies on sharing data and materials.” (as detailed online in our guide for authors http://journals.plos.org/plosone/s/competing-interests). If this adherence statement is not accurate and there are restrictions on sharing of data and/or materials, please state these. Please note that we cannot proceed with consideration of your article until this information has been declared.**

**Please include both an updated Funding Statement and Competing Interests Statement in your cover letter. We will change the online submission form on your behalf.**

**Response**

**The statement in the Funding Statement and Competing Interests Statement has been revised as follows.**

**Funding Statement and Competing Interests Statement**

**This work was supported by the Dental Support Co., Ltd. and a JSPS KAKENHI (Grant**

**No. 25463029). Takeshi Uchida and Ikuo Kantake are employed by Dental Support Co. Ltd. Takeshi Uchida played roles in validation and resourcing for this study and Ikuo Kantake played roles in Funding acquisition for this study. The remaining authors declare that the research was conducted in the absence of any commercial or financial relationships that could be construed as a potential conflict of interest. Funders provided support in the form of salaries for authors, but did not have any additional role in the study design, data collection and analysis, decision to publish, or preparation of the manuscript. The specific roles of these authors are articulated in the ‘author contributions’ section. This does not alter our adherence to PLOS ONE policies on sharing data and materials.**

**4. When completing the data availability statement of the submission form, you indicated that you will make your data available on acceptance. We strongly recommend all authors decide on a data sharing plan before acceptance, as the process can be lengthy and hold up publication timelines. Please note that, though access restrictions are acceptable now, your entire data will need to be made freely accessible if your manuscript is accepted for publication. This policy applies to all data except where public deposition would breach compliance with the protocol approved by your research ethics board. If you are unable to adhere to our open data policy, please kindly revise your statement to explain your reasoning and we will seek the editor's input on an exemption. Please be assured that, once you have provided your new statement, the assessment of your exemption will not hold up the peer review process.**

**Response**

**The authors confirm that all data underlying the findings described in their manuscript are fully available without restriction.**

**Reviewers' comments:

Reviewer's Responses to Questions**

**1. Is the manuscript technically sound, and do the data support the conclusions?

The manuscript must describe a technically sound piece of scientific research with data that supports the conclusions. Experiments must have been conducted rigorously, with appropriate controls, replication, and sample sizes. The conclusions must be drawn appropriately based on the data presented.**

**Reviewer #1: Yes**

**Reviewer #2: Yes**

**2. Has the statistical analysis been performed appropriately and rigorously?**

**Reviewer #1: Yes**

**Reviewer #2: Yes**

**3. Have the authors made all data underlying the findings in their manuscript fully available?

The** [**PLOS Data policy**](http://www.plosone.org/static/policies.action#sharing) **requires authors to make all data underlying the findings described in their manuscript fully available without restriction, with rare exception (please refer to the Data Availability Statement in the manuscript PDF file). The data should be provided as part of the manuscript or its supporting information, or deposited to a public repository. For example, in addition to summary statistics, the data points behind means, medians and variance measures should be available. If there are restrictions on publicly sharing data—e.g. participant privacy or use of data from a third party—those must be specified.**

**Reviewer #1: Yes**

**Reviewer #2: Yes**

**4. Is the manuscript presented in an intelligible fashion and written in standard English?

PLOS ONE does not copyedit accepted manuscripts, so the language in submitted articles must be clear, correct, and unambiguous. Any typographical or grammatical errors should be corrected at revision, so please note any specific errors here.**

**Reviewer #1: Yes**

**Reviewer #2: Yes**

**5. Review Comments to the Author

Please use the space provided to explain your answers to the questions above. You may also include additional comments for the author, including concerns about dual publication, research ethics, or publication ethics. (Please upload your review as an attachment if it exceeds 20,000 characters)**

**Reviewer #1: The manuscript was easy to read and easy to understand. My main concern is with the sample size.**

**fNIRS should be mentioned in the title (+/-) abstract.**

**Response**

**Thank you for pointing this out. According to your suggestion, we added the “use of fNIRS” in the Abstract (p2 l8).**

**Even though the oral cavity is not visible, food chewing can be performed without damaging the tongue, oral mucosa, or other intraoral parts, with cross-modal perception of chewing possibly critical for appropriate recognition of its performance. This study was conducted to clarify the relationship of chewing food cross-modal perception with cortex activities based on examinations of the posterior parietal cortex (PPC) and visual cortex during chewing in comparison with sham chewing without food, imaginary chewing, and rest using functional near-infrared spectroscopy (fNIRS). Additionally, the effects of a deafferent tongue dorsum on PPC/visual cortex activities during chewing performance were examined. The results showed that chewing food increased activity in the PPC/visual cortex as compared with imaginary chewing, sham chewing without food, and rest, whereas that activity conducted with imaginary chewing and sham chewing without food was not significantly different as compared with rest. Moreover, subjects with a deafferent tongue dorsum showed reduced PPC/visual cortex activities during chewing food performance. Together, these results suggest that representation of PPC/visual cortex activities while chewing food suggests cross-modal recognition of chewing, while an oral somatosensory deficit may modulate cross-modal PPC/visual cortex activities during chewing performance.**

**Methods: "Since the necessary minimum sample size was shown to be three..." Please report more details (options and parameters chosen in GPower 3) that led to this calculation. It looked strange, as very few neuroimaging papers nowadays advocate such a small sample size.**

**Response**

**We appreciate the reviewer for pointing this out. In the original version, the number of measurements was incorrect. We were mistaken about the number of measurements. That has been corrected to 25 measurements (5 times repeated, 5 tasks) and the sample size was then recalculated using the following parameters: effect size = 0.3, α = 0.05, power (1-β) = 0.95, number of groups = 1, number of measurements = 25 (5 times repeated, 5 tasks). The sample size required was 9 and the power of this calculation was determined to be 0.974. The modified the section of the Subjects (p4 l64) and the explanation regarding sample size has been added to the Discussion section (p18 l372) in revised version.**

**(p4 l64)**

**The sample size was then recalculated using the following parameters: effect size = 0.3, α = 0.05, power (1-β) = 0.95, number of groups = 1, number of measurements = 25 (5 times repeated, 5 tasks). The sample size required was 9 and the power of this calculation was determined to be 0.974 [22].**

**(p18 l372)**

**Although, for the present study, two-way analysis of variance was used when both of the factors were corresponding. All factors were within-subject factors, which resulted in a greater number of measurements (25 times per subject). This within-subjects design has advantages, including fewer subjects required, minimized random noise, easier to find true differences between conditions, and increased power of the test [94-96]. The power calculation for nine participants is shown following: Power (1-β) = 0.974 [effect size = 0.3, power (β/α ratio) = 0.95, total sample size = 9, number of groups = 1, number of measurements = 25 (5 times repeated, 5 task)], and is considered to be sufficient.**

**[94] Charness G, Gneezy U, Kuhn MA. Experimental methods: Between-subject and within-subject design. Journal of Economic Behavior & Organization. 2012; 81: 1–8. doi: 10.1016/j.jebo.2011.08.009**

**[95] Bellemare C, Bissonnette L, Kröger S. Statistical Power of Within and Between-Subjects Designs in Economic Experiments. Institute for Labor Economics (IZA), Discussion Paper no. 8583.**

**[96] Montoya AK. Selecting a Within- or Between-Subject Design for Mediation: Validity, Causality, and Statistical Power. Multivariate Behav Res. 2023; 58: 616-636. doi: 10.1080/00273171.2022.2077287.**

**P11 L219 & P12 L237: "元文" should be removed.**

**Response**

**Thanks for pointing out the mistake, we removed "元文".**

**Reviewer #2: Title: Cross-modal representation of chewing food in posterior parietal and visual cortex

The current study focuses on an interesting subject of cross-modal representation during food chewing. However, to enhance the manuscript, several modifications are recommended.**

**Introduction:
1. The current introduction is somewhat limited. To underscore the significance of the study, additional information about clinical implications should be incorporated.**

**Response**

**Thank you for pointing this out. We have included additional information regarding clinical implications in the Introduction section (p3 l32) in accordance with your suggestion.**

**If a relationship of oral register discrimination with PPC/visual cortex activities exists, then masticatory function for forming a food mass without damaging the oral cavity during mastication may also require cross-modal communication of oral somatosensory and visual sensations, resulting in those activities being shown during mastication. Based on speculation that PPC/visual cortex activities are exhibited during mastication, the present study was conducted to further investigate the association between oral somatosensory and those activities during chewing food performance by contrasting chewing food with sham chewing without food, imaginary chewing, and rest conditions.**

**2. I recommend including relevant literature using other neuroimaging tools and elucidating the rationale behind choosing fNIRS for the current investigation.**

**Response**

**Thank you for pointing this out. We have followed your suggestion and included the reasons for selecting fNIRS in the Introduction section (p4 l49).**

**In the present study, fNIRS was applied for several reasons, including 1) its previous use for evaluations of those activities in regard to oral discrimination, 2) application for examination of mastication tasks in a normal setting, and 3) ease of establishing the experimental environment and use by the subjects. In the present study, fNIRS was employed to investigate manifestations of cross-modal associations of oral somatosensory-PPC/visual cortex activities during food chewing. Furthermore, the results obtained may show physiological and pathological cross-modality associations between oral somatosensory food percepts and PPC/visual cortex activities involved in recognition of chewing food during chewing.**

**3. The inclusion of a clear statement of the hypothesis is recommended to provide a clear framework for the study.**

**Response**

**Thank you for pointing this out. I have followed your suggestion and clearly stated the hypothesis in the Introduction section (p3 l44).**

**It is considered that findings indicating cross-modality of masticatory food masses would be helpful to clarify the direct relationship between tactual and visual cross-modal food recognition of those food masses as well as masticatory performance, in addition to the previously reported relationship between oral shape perception and masticatory performance.**

**Methods:
1. The variables considered for power analyses need to be explicitly stated for transparency.**

**Response**

**We appreciate the reviewer for pointing this out. In the original version, the number of measurements was incorrect. We were mistaken about the number of measurements. That has been corrected to 25 measurements (5 times repeated, 5 tasks) and the sample size was then recalculated using the following parameters: effect size = 0.3, α = 0.05, power (1-β) = 0.95, number of groups = 1, number of measurements = 25 (5 times repeated, 5 tasks). The sample size required was 9 and the power of this calculation was determined to be 0.974. The modified the section of the Subjects (p4 l64) and the explanation regarding sample size has been added to the Discussion section (p18 l372) in revised version.**

**(p4 l64)**

**The sample size was then recalculated using the following parameters: effect size = 0.3, α = 0.05, power (1-β) = 0.95, number of groups = 1, number of measurements = 25 (5 times repeated, 5 tasks). The sample size required was 9 and the power of this calculation was determined to be 0.974 [22].**

**(p18 l372)**

**Although, for the present study, two-way analysis of variance was used when both of the factors were corresponding. All factors were within-subject factors, which resulted in a greater number of measurements (25 times per subject). This within-subjects design has advantages, including fewer subjects required, minimized random noise, easier to find true differences between conditions, and increased power of the test [94-96]. The power calculation for nine participants is shown following: Power (1-β) = 0.974 [effect size = 0.3, power (β/α ratio) = 0.95, total sample size = 9, number of groups = 1, number of measurements = 25 (5 times repeated, 5 task)], and is considered to be sufficient.**

**[94] Charness G, Gneezy U, Kuhn MA. Experimental methods: Between-subject and within-subject design. Journal of Economic Behavior & Organization. 2012; 81: 1–8. doi: 10.1016/j.jebo.2011.08.009**

**[95] Bellemare C, Bissonnette L, Kröger S. Statistical Power of Within and Between-Subjects Designs in Economic Experiments. Institute for Labor Economics (IZA), Discussion Paper no. 8583.**

**[96] Montoya AK. Selecting a Within- or Between-Subject Design for Mediation: Validity, Causality, and Statistical Power. Multivariate Behav Res. 2023; 58: 616-636. doi: 10.1080/00273171.2022.2077287.**

**2. Inclusion and exclusion criteria should be clearly outlined to enhance the study's transparency.**

**Response**

**In accordance with your suggestion, we have revised the description of subjects to clearly state the inclusion and exclusion criteria (p5 l68).**

**The inclusion criteria used for the healthy subjects were as follows: 1) no symptoms indicating temporomandibular joint or masticatory muscle dysfunction in examinations conducted using diagnostic criteria for temporomandibular disorders [23]; 2) mentally healthy, as indicated by a score of <7 on the Hospital Anxiety and Depression Scale [24]; 3) no abnormalities found in a cranial examination; and 4) not currently taking any medication. Exclusion criteria for healthy subjects included: 1) presence of temporomandibular joint or masticatory muscle dysfunction; 2) mental impairment, as indicated by a score of >7 on the Hospital Anxiety and Depression Scale [24]; and 3) presently taking medication.**

**3. Demographic information about participants, including gender and ethnicity, should also be incorporated.**

**Response**

**As noted by the reviewer, the ethnicity of the subjects has been included in the revised version (p4 l62).**

**Nine right-handed healthy adult Asian males aged 23 to 47 years (30.8 ± 8.8 years, mean ± SD) participated in this study, with hand dominance confirmed using the Edinburgh Handedness Inventory [21].**

**4. Did the author apply any methods to ensure consistent probe set placements across participants, for example, the use of facial landmarks?**

**Response**

**Facial landmarks were not used, but the probe was placed in the center of the bottom line of the shell placed on the inion (Iz) according to the international 10-20 system, so that the probe was placed in the same position in each subject. The distance between the tip of the probe and the bottom of the shell was 10 mm.**

**5. Given that some studies found better correlations between fNIRS-related deoxygenated hemoglobin concentration and fMRI findings, it is advisable to reconsider the rationale for utilizing Oxy versus Deoxy values in the current study.**

**Response**

**We appreciate this helpful suggestion. Additional information and literature references regarding the basis for using oxy and deoxy values have been included in the revised version (p10 l184).**

**Oxygenated hemoglobin (Oxy-Hb) signal results have been widely reported in clinical research studies [49], with findings indicating better sensitivity to task-related hemodynamic changes [50] and excellent reliability for task-related activities [51]. Based on our previous results [52-54], Oxy-Hb signals were focused on in the present study.**

**[49] Ozawa S. Application of Near-Infrared Spectroscopy for Evidence-Based Psychotherapy. Front Psychol. 2021;12:527335–527335. https:// doi. org/ 10. 3389/ fpsyg. 2021. 527335.**

**[50] Yeung MK, Lin J. Probing depression, schizophrenia, and other psychiatric disorders using fNIRS and the verbal fluency test: A systematic review and meta-analysis. J Psychiatr Res. 2021;140:416–35. https:// doi. org/ 10. 1016/j. jpsyc hires. 2021. 06. 015.**

**[51] Huang Y, Mao M, Zhang Z, Zhou H, Zhao Y, Duan L. Test-retest reliability of the prefrontal response to affective pictures based on functional nearinfrared spectroscopy. J Biomed Opt. 2017;22(1):16011–23. https:// doi. org/ 10. 1117/1. JBO. 22.1. 016011.**

**[52] Narita N, Kamiya K, Iwaki S, Ishii T, Endo H, Shimosaka M, Uchida T, Kantake I, Shibutani K. Activation of Prefrontal Cortex in Process of Oral and Finger Shape Discrimination: fNIRS Study. Front Neurosci. 2021 Feb 5;15:588593. doi: 10.3389/fnins.2021.588593.**

**[53] Narita N, Ishii T, Iwaki S, Kamiya K, Okubo M, Uchida T, Kantake I, Shibutani K. Prefrontal Consolidation and Compensation as a Function of Wearing Denture in Partially Edentulous Elderly Patients. Front Aging Neurosci. 2020 Jan 31;11:375. doi: 10.3389/fnagi.2019.00375.**

**[54] Narita N, Kamiya K, Makiyama Y, Iwaki S, Komiyama O, Ishii T, Wake H. Prefrontal modulation during chewing performance in occlusal dysesthesia patients: a functional near-infrared spectroscopy study. Clin Oral Investig. 2019 Mar;23(3):1181-1196. doi: 10.1007/s00784-018-2534-7.**

**6. The data analysis pipeline should be explicitly described, encompassing filtering, removal of movement artifacts, and the model used to estimate fNIRS findings.**

**Response**

**Thank you for your advice. The fNIRS measurements session contains the following information (p10 l187).**

**The description of fNIRS data analysis has been moved from the fNIRS measurements section to a separate section (fNIRS data analysis) in the revised manuscript for clarity (p9 l176).**

**The sampling interval was 0.1 seconds. Each trial was repeated five times and obtained values were averaged using the ‘integral mode’ of the ETG-100 software for the Rest, Chewing, Image, Sham, and Anesthesia sessions. Also, a linear fitting algorithm [55] was used for baseline corrections [56]. A moving average with a window width of 5 seconds was used to remove physiological noise such as cardiac artifacts [57] and short-term motion artifacts [58,59] in the fNIRS signals.**

**Results:**

**1. Sections of the description from lines 220-224 and lines 238-244 appear repetitive and should be revised for conciseness.**

**Response**

**Thank you for pointing it out. We have deleted the part you mentioned, since it is doubly stated.**

**P11 l 220 ~ p12 l224**

**During the resting session, there were no obvious changes in [oxy-Hb] in the occipital lobe (Fig. 2A). In the Chewing session, distinct increases in [oxy-Hb] were observed in PPC (BA7), VAC (BA19), PSC (BA18), and SC 221 (BA17) (Fig. 2B). In the Image session, [oxy-Hb] increased in SC (BA17) during the task period (Fig. 2C). In the Sham session, there was an increase in [oxy-Hb] in PPC (BA7) and SC (BA17) during the task period (Fig. 2D). In the Anesthesia session, [oxy-Hb] increased in PPC (BA7), VAC (BA19), PSC (BA18) and SC (BA17) during the task period (Fig.2E).**

**P12 l238 ~ p13 l244**

**Left: Mean change in oxygenated hemoglobin concentration ([oxy-Hb], red line) and deoxygenated hemoglobin concentration ([deoxy-Hb], blue line) during Rest (A), Chewing (B), Imagery (C), Sham (D), and Anesthesia (E) sessions. X axis indicates time (s); Y axis indicates hemodynamic change ( mMmm); the gray vertical lines at 20 and 30 seconds indicate the beginning and end of the 10-second task period, respectively.**

**Right: Topographical maps showing changes in [oxy-Hb] for 10-s before task (Pre-task), 10-s during task (Task), and 10-s after task (Post-task). No obvious changes were observed in the Rest (A), but there was a significant increase in 243 the Chewing (B), a slight increase in the Imagery (C) and Sham (D), and a significant increase in the Anesthesia during the Task and Post-task (E).**

**2. While significant time x ROI interactions were identified, the temporal distinctions across each ROI in post-hoc analyses were not specified.**

**Response**

**Thank you for pointing it out. In this study, we added the description "during task" to the description of the results because we are not interested in finer differences than before, during, and after the task.**

**Comparison between Chewing and Rest**

**When Chewing was compared to Rest, the values for [oxy-Hb] were significantly (*p* < 0.05) increased during the task periods in PPC (Fig. 3A), PPC/VAC (Fig. 3B), VAC/PSC (Fig. 3C), PSC/SC (Fig. 3D), and SC (Fig.3E).**

**Comparison between Image and Rest**

**The values for [oxy-Hb] were not significantly different between Image and Rest during the task periods.**

**Comparison between Sham and Rest**

**The values for [oxy-Hb] were not significantly different between Sham and Rest during the task periods.**

**Comparison between 265 Anesthesia and Rest**

**The values for [oxy-Hb] were not significantly different between Anesthesia and Rest during the task periods.**

**Comparison between Chewing and Image**

**When Chewing was compared to Image, the values for [oxy-Hb] were significantly (p < 0.05) increased in PPC (Fig. 3A), PPC/VAC (Fig. 3B), VAC/PSC (Fig. 3C), PSC/SC (Fig. 3D), and SC during the task periods (Fig.3E).**

**Comparison between Chewing and Sham**

**When Chewing was compared to Sham, the values for [oxy-Hb] were significantly (p < 0.05) increased during the task periods in PPC (Fig. 3A), VAC/PSC (Fig. 3B), PSC/SC (Fig. 3D), and SC (Fig. 3E).**

**Comparison between Chewing and Anesthesia**

**When Chewing was compared to Anesthesia, the values for [oxy-Hb] were significantly (p < 0.05) increased during the task periods in VAC/PSC (Fig. 3C), PSC/SC (Fig. 3D), and SC (Fig. 3E).**

**Discussion:
1. It is essential to incorporate a discussion of study limitations, including the small sample size, and provide insights into potential future research directions.**

**Response**

**As suggested by the reviewers, the following Study limitations and Future research directions section has been added to the Discussion section and literature references regarding the clinical applications (p18 l366).**

**Study limitations and Future research directions**

**This study has several limitations. This study is the small sample size, which may not allow for more reliable results in this study. The sample size is not sufficient to draw conclusions about the general population. In the present study, healthy subjects were used as test subjects, but this is not a sufficient sample size considering the influence of oral conditions such as occlusal conditions and the effect of anesthesia on the tongue. A larger sample is needed to test the hypothesis. Although, for the present study, two-way analysis of variance was used when both of the factors were corresponding. All factors were within-subject factors, which resulted in a greater number of measurements (25 times per subject). This within-subjects design has advantages, including fewer subjects required, minimized random noise, easier to find true differences between conditions, and increased power of the test [94-96]. The power calculation for nine participants is shown following: Power (1-β) = 0.974 [effect size = 0.3, power (β/α ratio) = 0.95, total sample size = 9, number of groups = 1, number of measurements = 25 (5 times repeated, 5 task)], and is considered to be sufficient. Also, the spatial resolution of fNIRS is less than that of fMRI. The spatial resolution of fNIRS is limited by the number of detector optodes and the physics of light scatter from the emitter to the detector. The systems in this study uses detectors spaced ∼3 cm apart from an illuminating optodes that inform on brain activity along a curved path from the emitter to the detector, increasing the spatial uncertainty of the signal.**

**For clinical applications, as reported in this study, tactile and visual cross-modal representations that may be involved in perception of masticated food masses during chewing are perceived in the PPC/visual cortex. Mastication has often been functionally evaluated by examining the crushed state of masticated food [97,98], glucose extracted from masticated food [99,100], or color change of masticated food [101,102], as well as other factors. Furthermore, various approaches have been used to determine the relationship between masticatory function and oral shape awareness [103, 104]. Taken together, the results of those previous studies and the present suggest that activities of the PPC/visual cortex, which may be involved in perception of masticatory food masses, are related to food crushing, glucose release, and gum discoloration, and are affected by occlusal conditions and/or prosthetic treatment. Additional clinical studies of these factors will be helpful to provide greater elucidation of not only the association of mastication with oral stereognostic ability, but also the direct relationship between mastication and food mass perception ability.**

**[97] van der Bilt A, van der Glas HW, Mowlana F, Heath MR. A comparison between sieving and optical scanning for the determination of particle size distributions obtained by mastication in man. Arch Oral Biol. 1993; 38:159-162. doi: 10.1016/0003-9969(93)90201-v.**

**[98] Buschang PH, Throckmorton GS, Travers KH, Johnson G. The effects of bolus size and chewing rate on masticatory performance with artificial test foods. J Oral Rehabil. 1997; 24: 522-526. doi: 10.1046/j.1365-2842.1997.00524.x.**

**[99] Aiyar A, Shimada A, Svensson P. Assessment of masticatory efficiency based on glucose concentration in orthodontic patients: A methodological study. J Oral Rehabil. 2022; 49:954-960. doi: 10.1111/joor.13359.**

**[100] Shiga H, Nakajima K, Uesugi H, Komino M, Sano M, Arai S. Reference value of masticatory performance by measuring the amount of glucose extraction from chewing gummy jelly. J Prosthodont Res. 2022; 66: 618-622. doi: 10.2186/jpr.JPR_D_21_00154.**

**[101] Kamiyama M, Kanazawa M, Fujinami Y, Minakuchi S. Validity and reliability of a Self-Implementable method to evaluate masticatory performance: use of color-changeable chewing gum and a color scale. J Prosthodont Res. 2010; 54: 24-28. doi: 10.1016/j.jpor.2009.08.001.**

**[102] Takahara M, Shiraiwa T, Maeno Y, Yamamoto K, Shiraiwa Y, Yoshida Y, et al. Screening for a decreased masticatory function by a color-changeable chewing gum test in patients with metabolic disease. Intern Med. 2022; 61: 781-787. doi: 10.2169/internalmedicine.8082-21.**

**[103] Kumamoto Y, Kaiba Y, Imamura S, Minakuchi S. Influence of palatal coverage on oral function - oral stereognostic ability and masticatory efficiency. J Prosthodont Res. 2010; 54: 92-96. doi: 10.1016/j.jpor.2009.11.006.**

**[104] Mary KM, Cherian B. Evaluation of oral stereognosis, masticatory efficiency, and salivary flow rate in complete denture wearers. J Indian Prosthodont Soc. 2020; 20: 290-296. doi: 10.4103/jips.jips_453_19.**

**While revising your submission, please upload your figure files to the Preflight Analysis and Conversion Engine (PACE) digital diagnostic tool,** [**https://pacev2.apexcovantage.com/**](https://pacev2.apexcovantage.com/)**. PACE helps ensure that figures meet PLOS requirements. To use PACE, you must first register as a user. Registration is free. Then, login and navigate to the UPLOAD tab, where you will find detailed instructions on how to use the tool. If you encounter any issues or have any questions when using PACE, please email PLOS at** [**figures@plos.org**](mailto:figures@plos.org)**. Please note that Supporting Information files do not need this step.**

**Response**

**We used PACE to ensure that the charts met the PLOS requirements.**
