# Supplementary material for: Cross-modal representation of chewing food in posterior parietal and visual cortex
Source: PLoS One. 2024 Oct 25;19(10):e0310513. doi: 10.1371/journal.pone.0310513 (PMC11508057; doi:10.1371/journal.pone.0310513)
Supplement: Attachment — Submitted filename: Response to Reviewers.docx [file pone.0310513.s003.docx]

Response to Editor and Reviewers

Comment 1. Please review your reference list to ensure that it is complete and correct. Any changes to the reference list should be mentioned in the rebuttal letter that accompanies your revised manuscript.

Response

1. Reference [22] was removed since reference [22] was used as the evidence for the sample size. Therefore, the subsequent reference numbers will be moved up by one and the total number of references will be changed to 103.
2. Reference [69] was changed to the following reference because the Effect size was changed to f. We changed the reference from “69. Cohen, J. Statistical power analysis for the behavioral sciences. 2nd ed. Hillsdale, NJ: Lawrence Erlbaum; 1988”. to “69. Cohen, J. A power primer. Psychol Bull.1992; 112: 155-159. doi: 10.1037//0033-2909.112.1.155”.
3. The following three references were added to the Study limitation and future research directions section in the discussion.

93. Gail M. Sullivan, MD, MPH and Richard Feinn, PhD Using Effect Size—or Why the P Value Is Not Enough J Grad Med Educ. 2012 Sep; 4(3): 279–282. doi: 10.4300/JGME-D-12-00156.1

94. Julien KC, Buschang PH, Throckmorton GS, Dechow PC. Normal masticatory performance in young adults and children. Arch Oral Biol. 1996; 41: 69-75. doi: 10.1016/0003-9969(95)00098-4.

95. Månsson I, Sandberg N. Effects of surface anesthesia on deglutition in man. Laryngoscope. 1974; 84: 427-437. doi: 10.1288/00005537-197403000-00006.

Comment 2. Power analysis: Could you please clarify how the parameters of the new power analysis map onto the statistical tests which are reported in the results section? Number of measurements was set to 25 (“5 times repeated, 5 tasks”): Does “5 times repeated” refer to the 5 trials per task condition which were recorded? It is my understanding that the 5 trials were averaged for the purpose of the statistical analyses (p. 10 l. 188), therefore trial repetition is not a repeated measurement factor in the analysis and the number of measurements for the main effect of condition would be 5 (i.e., the 5 task conditions) rather than 25. Note that G*Power does not seem to allow calculation of power for an interaction effect between two within-subject factors (such as Condition x Time Point), which might in fact require a simulation approach. For the main effect of condition, the reported power analysis in G*Power would yield a required sample size of n=22 to show an effect of size f=0.3 if the number of measurements were set to 5 (instead of 25). As an alternative to reporting an a priori power analysis, a sensitivity analysis could be reported, which would indicate that the sample size of n=9 had 95% power (with number of measurements set to 5) to show an effect of size f=0.49 (i.e., a large effect according to your definition on p. 12).

Response

We sincerely appreciate your advice. According with your suggestion, we have changed to report the results of the sensitivity analysis calculation in the results section, instead of the sample size calculation. Accordingly, we have made the following changes.

1. Removed sentence regarding sample size “The sample size was then recalculated using the following parameters: effect size = 0.3, α = 0.05, power (1-β) = 0.95, number of groups = 1, number of measurements = 25 (5 times repeated, 5 tasks). The sample size required was 9 and the power of this calculation was determined to be 0.974 [22]” in Materials and methods section.
2. The sentence of effect size was changed from “Eta squared (η2) and r were used to estimate effect size. The effect size was used to estimate the standardized degree of effect, including differences, influence, correlations, and association of data. For effect size (η2), the magnitude of effect was defined as 0.01 = small, 0.06 = medium, and 0.14 = large, while for effect size (r), the magnitude of effect was defined as 0.1 = small, 0.3 = medium, and 0.5 = large [70].” to “Cohen’s effect size (f) values were accessed for ANOVA results. Effect size was used to estimate the standardized degree of effect, including differences, and influence of the findings shown by the obtained data. Critical Cohen's f values for distinguishing small, moderate, and large effects were 0.10, 0.25, and 0.40, respectively [69].” in Statistical analysis section,
3. The sentence of “The numbness VAS scale value for all subjects before anesthesia was 0. A significant increase to 50.4 ± 0.4 (one-sample signed rank test, p = 0.004, r = 0.89) was noted after starting anesthesia.” changed to “The numbness VAS scale value for all subjects before anesthesia was 0. A significant increase to 50.4 ± 0.4 (one-sample signed rank test, p = 0.004) was noted after starting anesthesia.” in Numbness on tongue dorsal surface before and after anesthesia section of results.
4. The sentence of “During the Rest, Chewing, Image, Sham, and Anesthesia sessions, parietal and occipital lobe activities showed significant interactions with time in the PPC (CH 1, 2, 3) (F = 1.399 p < 0.001, η2 = 0.10), PPC/VAC (CH 4, 5, 6, 7) (F = 1.428 p < 0.001, η2 = 0.11), VAC/PSC (CH 8, 9, 10, 11, 14) (F = 1.527 p < 0.001, η2 = 0.11), PSC/SC (CH , 12, 13, 18, 21, 22, 24) (F = 2.779, p < 0.001, η2 = 0.16), and SC (CH 15, 16, 17, 19, 20, 23) (F = 3.312, p < 0.001, η2 = 0.19).” changed to “During the Rest, Chewing, Image, Sham, and Anesthesia sessions, parietal and occipital lobe activities showed significant interactions with time in the PPC (CH 1, 2, 3) (F = 1.399 p < 0.001, power = 0.943, f = 0.482), PPC/VAC (CH 4, 5, 6, 7) (F = 1.428 p < 0.001, power = 0.962, f = 0.505), VAC/PSC (CH 8, 9, 10, 11, 14) (F = 1.527 p < 0.001, power = 0.993, f = 0.587), PSC/SC (CH , 12, 13, 18, 21, 22, 24) (F = 2.779, p < 0.001, power = 1.00, f = 0.662), and SC (CH 15, 16, 17, 19, 20, 23) (F = 3.312, p < 0.001, power = 1.00, f = 0.662).” in Temporal changes in [oxy-Hb] during Rest, Chewing, Image, Sham, and Anesthesia section of results,

Comment 3. Abstract l. 15: Change “Together, these results suggest that representation of PPC/visual cortex activities while chewing food suggests…” to “Together, the result of PPC/visual cortex activities while chewing food suggests…”.

Response

We sincerely appreciate your advice. According with your suggestion, we corrected “Together, these results suggest that representation of PPC/visual cortex activities while chewing food suggests…” to “Together, the result of PPC/visual cortex activities while chewing food suggests”.

Comment 4. p. 4 l. 54-58: The last two sentences of the introduction appear somewhat redundant and could be condensed into one sentence.

Response

We sincerely appreciate your advice. According with your suggestion, we changed “This novel study was conducted to reveal the effects of chewing food perception on PPC/visual cortex activities during chewing performance, with discussion from the viewpoint of cross-modal food recognition while chewing” to “This novel study then used those results to reveal the effects of chewing food perception on PPC/visual cortex activities during chewing performance. Additionally, discussion from the viewpoint of cross-modal food recognition while chewing is presented”.

5. p. 18 l. 367ff.: There appears to be some redundancy in this paragraph which could be streamlined.

Response

We sincerely appreciate your advice. According with your suggestion, we changed Study limitations and Future research directions section in Discussion to “In the present study, results showing neural activity in the parietal and occipital lobes indicated a statistically significant interaction between task and time. Chewing food trials have found significant neural activation in the parietal and occipital lobes as compared to resting task trials, based on obtained P-values and effect sizes [93]. Effect size represents the magnitude of change in an outcome and often can be more important than just relying on P-values when interpreting study results, as that indicates the actual magnitude of difference. On the other hand, the present findings indicate that oral sensory deprivation results in significantly decreased neural activity in the parietal and occipital lobes during food chewing. Thus, it is considered that the requirement of representation of somatosensory-visual cross-modality for food bolus perception during chewing was confirmed. Nevertheless, in consideration of the variety of individual normal occlusion conditions encountered [94] as well as the influence of surface anesthesia in the mouth [95], the number of measurements in healthy subjects should be increased in future clinical trials, which may be a limitation of the present study.

Previous studies functionally evaluated chewing ability based on particle size distribution in comminuted test food [96, 97], findings following glucose extraction from chewed food [98, 99], and color changes in chewing gum used for testing [100, 101]. In addition, the relationship [102, 103] of chewing efficiency with oral stereognosis ability has also been examined in young healthy and edentulous subjects. In future clinical studies, measurements of PPC/visual cortex activities during chewing can be applied to evaluate chewing ability from the viewpoints of tactile and visual cross-modal representations of chewing food in the dentistry patients with chewing difficulties.
