# Supplementary material for: Cross-modal representation of chewing food in posterior parietal and visual cortex
Source: PLoS One. 2024 Oct 25;19(10):e0310513. doi: 10.1371/journal.pone.0310513 (PMC11508057; doi:10.1371/journal.pone.0310513)
Supplement: Attachment — Submitted filename: Response to Reviewers.docx [file pone.0310513.s004.docx]

Journal Requirements

Please review your reference list to ensure that it is complete and correct. If you have cited papers that have been retracted, please include the rationale for doing so in the manuscript text, or remove these references and replace them with relevant current references. Any changes to the reference list should be mentioned in the rebuttal letter that accompanies your revised manuscript. If you need to cite a retracted article, indicate the article’s retracted status in the References list and also include a citation and full reference for the retraction notice.

Response

Thank you for the suggestion. We have added the references, checked the citation order, and corrected the error.

Response to Editor

Comment 1.

Thank you for addressing my previous comments. However, I believe that the concerns regarding the power analysis have still not been fully resolved. It appears that what is now reported in the revised manuscript is a post-hoc power analysis, i.e. power was calculated based on the effect size that was observed in your study. Please note that reporting post-hoc power is usually considered pointless, as it does not add any information beyond the reported p values. Instead, a sensitivity power analysis should be performed as recommended in my comments on the previous version of the paper. A good discussion of the distinction between different types of power analyses and when to use them can, for example, be found in: Lakens (2022). Sample size justification. Collabra: Psychology 8(1). https://doi.org/10.1525/collabra.33267

Response

Thank you for your suggestion. I am very thankful that you even provided references. We have added a description of the sensitivity analysis in the subjects section (p4, l59). In addition, in the Results section, we have included an effect size for the 2-way ANOVA results and described the comparison to the minimum effect size obtained in the sensitivity analysis (p12, l234). In the Discussion section, in the Limitation section, we have modified the description of the validity of the 9 subjects and its limitations (p16, l329).

Comment 2.

　　On a related point: changing the reported effect sizes from eta squared to Cohen’s f values seems like a bad choice. Although both carry equivalent information and can be back-converted, eta squared is more commonly reported in ANOVAs and will thus be easier to interpret for readers.

Response

Thank you for your suggestion. We have combined η^2^ and f for the effect size in the description of the 2-way ANOVA results (p10, l194).
